# Problem-based Learning to Encourage Active Learning and Teamwork Among First Year Medical Students - Student Reports in 2021 - What is the Best Western Music to Listen to When Studying? (Course Name: Listening Skills: Development and Assessment)

**DOI:** 10.14789/jmj.JMJ22-0007-OT

**Published:** 2022-11-18

**Authors:** RYOTA OYA, MIHIRO SHIRATAKI, SHUJI MATSUMOTO, RAN YAMAGUCHI, RYOKO FUJITA

**Affiliations:** 1 School of Medicine, Juntendo University, Tokyo, Japan; 1 School of Medicine, Juntendo University, Tokyo, Japan; 2 Department of General Education, Juntendo University Faculty of Medicine, Chiba, Japan; 2 Department of General Education, Juntendo University Faculty of Medicine, Chiba, Japan

**Keywords:** music, learning style, listening, study environment

## Abstract

**Objective:**

The goal of this study was to evaluate how listening to different types of music while studying affects learning.

**Methods and Materials:**

We conducted a survey to discover people's music listening habits. We designated calculation tasks or memorization tasks and asked students to work on the tasks while listening to nothing or listening to music The types of Western music had three categories: the accent of the singer, the pitch, and the speed. The participants were divided into six groups based on what tasks they did and what types of music they listened to.

**Results:**

There was no correlation between the preference of study environment, whether students usually listen to Western music or any music while studying, and the task scores. We found that there was not much difference between the scores of calculations when listening to nothing or listening to some kind of music.

**Conclusions:**

It seems that the ideal type of music a student should listen to depends on what they study. It might be a good idea to listening to slow music or music at a low pitch when doing calculation tasks. On the other hand, when doing memorization tasks, it might be a good idea to play music that you have never listened to before.

## Introduction

We were curious about how listening to music while studying would affect memorization or calculation. We conducted an experiment on listening to music while studying and then analyzed and discussed the data. In this experiment, the music was limited to Western music. Various elements of music that might have influence on learning tasks were examined.

## Literature review

Previous studies have examined how music affects one's learning efficiency. Abe and Shingaki (2010) state that tasks such as calculation and inputting information on a PC, which require attention and are stressful to our brain, are not easily affected by the tempo of the background music. On the other hand, tasks that do not require much attention and are less stressful, such as simply walking down the road or doing housework, are easily affected by the tempo of background music^1)^.

Suga and Goto (2008) note that introducing background music when studying helps students psychologically rather than in a cognitive sense^2)^. The students felt more relaxed when listening to the music, but they were not able to concentrate on studying as they got distracted by the music.

Finally, Tsuyama and Takeyoshi (2015) conducted a series of experiments comparing primary schoolers, junior high schoolers and college students. The results showed that junior high schoolers' and college students' performance was the highest when there was no background music, and primary schoolers performed the best when listening to classical music. From this study, we can conclude that the most effective background music differs from age to age^3)^. The past studies mentioned above examined the effects of music on learning from various viewpoints, such as task types, psychological aspects, and age groups. However, the past studies did not examine the effects of music types and learners' habits of listening to music. Therefore, the following research questions (RQs) were developed for the current study.

RQ1: How do different types of music affect learners doing memorization tasks and the calculation tasks?

RQ2: Do learners' habit of listening to music while studying affect the memorization and calculation tasks while listening to music?

## Method

**Figure 1:**
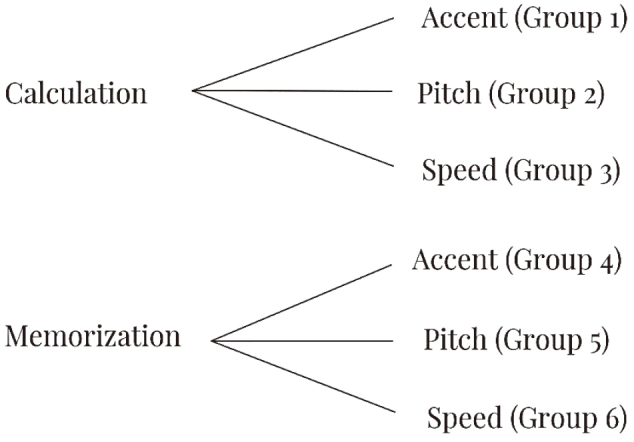
Outline of the experiments

**Figure 2:**
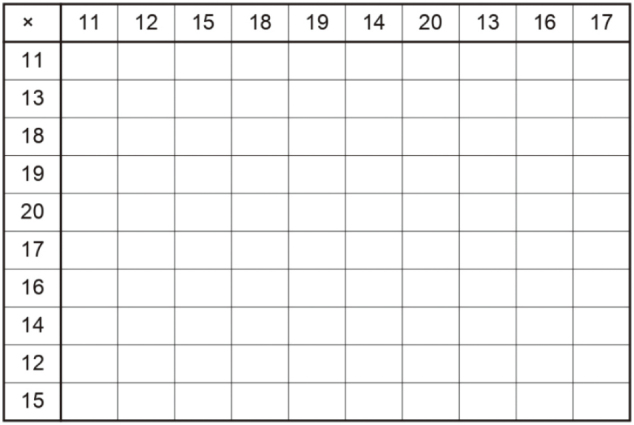
Example of the calculation task

**Figure 3:**
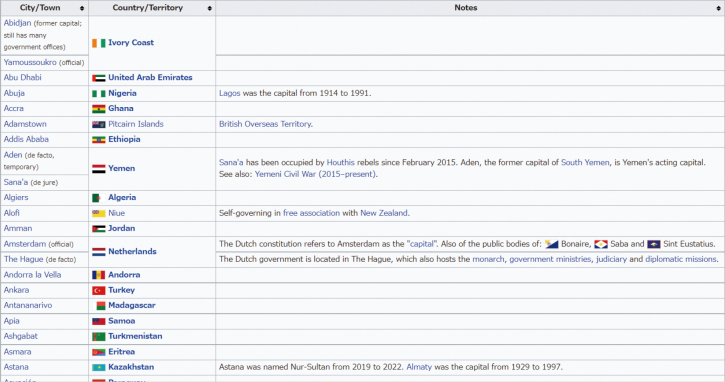
Example of the memorization task^4)^

Forty-three participants, who were first-year students from Juntendo Medical School, participated in the study. The participants worked on the assigned tasks: calculation and memorization (see Figure 1). We developed calculation tasks, which included 100 multiplications of two digits multiplied by two digits (see Figure 2). The memorization task asked the participants to look at a list of the world's capital cities and memorize as much as they could in two minutes^4)^ (see Figure 3). In this experiment, the participants were divided into six groups based on what tasks they did and what types of music they listened to. We refer to “calculation and accent” as Group 1, “calculation and pitch” as Group 2, “calculation and speed” as Group 3, “memorization and accent” as Group 4, “memorization and pitch” as Group 5 and “memorization and speed” as Group 6.

In each test, the same tasks were carried out first without listening to any music and then (second and third) while listening to two opposite types of music: for example, Indian English music and English music. It was preferrable to use different tasks in order to avoid practice effects, but due to the time constraint, the same tasks were used. We gathered data on the scores, the time it took to finish all 100 multiplications and the number of capital cities the participants were able to memorize. The types of Western music were grouped by three categories: the accent of the singer, the pitch and the speed. For the accent of the singer, Indian English music and English music were used. For Indian music, “Kya Soorat Hai” by an Indian pop group, *Bombay Vikings* was used. For English music, “What Makes You Beautiful” by an English pop group, *One Direction* was used. Two songs were used in the pitch task. “Aria the Queen of the Night” as a high-pitched song and “In this holy temple” as a low-pitched song, were selected from the opera *The Magic Flute*. To examine the speed, a rap song, “The search” by an American rapper, NF, was used as a fast song, and “My Heart Will Go On” by a Canadian singer, Celine Dion, was used as a slow song.

Furthermore, we asked the participants to answer a questionnaire about their music listening habits. The questionnaire items included how often they listened to Western music, how often they listened to any kind of music while studying and whether they knew the music used in this experiment.

## Results

### Calculation task

First, we analyzed the data from the calculation task. The main focus of the calculation task was the time that it took for the participants to finish the calculation task, rather than the correct answer rate. Therefore, we measured the amount of time it took for the participants to finish the task. Table 1 shows the results of Group 1, who worked on Calculation and accent task. In Group 1, Student (S) 1, S2, and S6 showed almost the same performance no matter what they listened to. The other two, S3 and S4 did better when listening to music. The others, S5 and S7 performed best without music.

**Table 1:** Results of Group 1: Calculation and accent

Group 1	No Music[seconds]	Native English[seconds]	Indian English[seconds]
S1	220	207	218
S2	167	188	144
S3	1004	744	758
S4	900	780	600
S5	215	495	593
S6	585	579	590
S7	454	537	502
*M*	506.4	504.3	486.4
*SD*	340.0	234.0	223.0

Note: *N* = 7. S1 = student 1.

Table 2 shows the results of the Group 2 who finished Calculation and pitch task. In group 2, four out of seven, S8, S10, S11, and S15 seemed not be affected by music when doing calculation tasks. On the other hand, S12, S13, and S14 did best when they listened to Low Pitch music. Besides, these three participants shortened the length of time as they repeated the test. What was interesting about this group is that only S9 showed best performance without music.

**Table 2:** Results of Group 2: Calculation and pitch

Group 2	No Music[seconds]	Native English[seconds]	Indian English[seconds]
S8	412	421	432
S9	532	689	634
S10	210	191	203
S11	142	158	160
S12	327	294	274
S13	612	443	420
S14	825	735	710
S15	470	440	433
*M*	441.3	421.4	408.3
*SD*	221.2	210.3	194.6

Note: *N* = 8. S8 = student 8. [s] = seconds.

Table 3 shows the results of Group 3, who participated in the Calculation and speed task. Three out of five participants, which are No. S16, S17, and S20, took almost the same length of time in three tasks. S18 shortened time as the test is repeated. S19 also performed best in the last test. However, the participants relatively took the longest time in the second task, which as fast music.

**Table 3:** Results of Group 3: Calculation and speed

Group 3	No Music[seconds]	Native English[seconds]	Indian English[seconds]
S16	167	229	181
S17	220	194	206
S18	923	763	548
S19	483	527	360
S20	543	552	545
*M*	467.2	453.0	368.0
*SD*	302.1	239.1	176.8

Note: *N* = 5. S16 = student 16. [s] = seconds.

For Groups 1 to 3, there was not much difference between the scores when listening to nothing or listening to some kind of music. Nevertheless, we could see that there were differences among individuals. We also asked whether they listened to Western music daily and whether they listened to music when they studied. We checked whether there was any correlation between the individual results and the answers to these two questions, but we could not find any. In addition, the results did not show any significant differences between the two songs. In conclusion, calculation tasks are not affected by the presence or absence of background music.

### Memorization task

We analyzed the data from the memorization task. Unlike the calculation task, the main focus of the memorization task was how many capital cities the participants were able to memorize in two minutes. Therefore, we counted the number of capital cities that they were able to answer correctly. Table 4 shows the result of Group 4 who participated in the Memorization and accent task. In Group 4, two of the seven students, S21 and S25 performed well on the English song, and four, S22, S24, S26, and S27, performed well on the Indian English song. There was not much of a difference between the two songs, but interestingly, most of the respondents said that Indian English music was easier to work on.

**Table 4:** Group 4: Memorization and accent

Group 4	No Music	Native English	Indian English
S21	9	13	11
S22	11	8	12
S23	1	1	1
S24	4	4	7
S25	9	10	4
S26	7	4	7
S27	9	8	12
*M*	7.1	6.9	7.7
*SD*	3.5	4.1	4.2

Note: *N* = 7. S21 = student 21.

As shown in Table 5, Group 5 worked on the Memorization and pith task. All the participants but S11 suffered greatly from listening to music. S29 and S31's memory increased when listening to low-pitched music, while S28, S30, and S34's performances were better when listening to high-pitched music. The ease of memorization due to the difference in pitch varied from person to person. However, it turned out that listening to high-pitched music can help them memorize more.

**Table 5:** Results of Group 5: Memorization and pitch

Group 5	No Music	High Pitch	Low Pitch
S28	9	5	4
S29	10	4	6
S30	21	17	11
S31	6	4	6
S32	9	4	4
S33	11	15	9
S34	10	8	6
*M*	10.9	8.1	6.6
*SD*	4.7	5.6	2.6

Note: *N* = 7. S28 = student 28.

Table 6 shows the results of the last group, Group 6, who did the memorization task while listening to a rap and a ballade. Results of S35, S36, S37, S39, and S42 indicates that listening to music while memorizing makes it difficult to memorize, compared to listening to nothing, since the number of capital cities they were able to memorize was the largest in the no music condition. It should be noted that S36 was exceptional since S36's memorization ability was high and he was able to memorize incredible number of capitals in all the conditions.

**Table 6:** Results of Group 6 Memorization and speed

Group 6	No music	Fast music	Slow music
S35	10	5	6
S36	37	35	21
S37	14	7	9
S38	6	3	7
S39	11	8	10
S40	3	5	8
S41	7	8	8
S42	17	14	6
S43	4	8	5
*M*	12.1	10.3	8.9
*SD*	10.4	9.7	4.8

Note: *N* = 9. S35 = student 35.

To summarize, for Groups 4 and 6, we could not find any common tendency between the results obtained while listening to nothing or listening to music. However, we found different tendencies in terms of accent for Group 4 and speed for Group 6. The reason we could not find any common tendencies was because the results differed greatly among individuals. However, six out of seven people in Group 4 answered that they felt it easier to memorize while listening to Indian English music compared to Native English music. For Group 5, the results came back generally better when listening to no music. When we compared between high-pitched and low-pitched songs, high-pitched songs showed better results. We could not find any correlation between the scores and the two questionnaire items mentioned above.

## Discussion and Conclusion

Judging from the results, the best circumstance when studying might be to study without any background music because the participants in the current study generally scored better under the no-background music condition. However, if the noise in someone's environment is annoying and requires them to listen to some music, it seems that the ideal type of music people might want to listen to depends on what they study. According to the results, it was suggested that playing slow music or music at a low pitch when doing calculation tasks might be good because the participants were able to finish the calculation task faster under the conditions in the low pitch song and also the slow music conditions than those under counterpart conditions. These results do not support what Abe and Shingaki (2010) argued^1)^. They argued that tasks which require attention and stress to our brain are not easily affected by the tempo of the background music, which implies that people concentrating on the calculation task do not easily get affected by the tempo of the background music. The results of the current study, however, showed that playing slow music or music at a low pitch might be good for calculation tasks. These results showed that it is difficult to generalize the best type of music to listening to for calculation tasks.

When doing memorization tasks, it may be best to play music they have never listened to before because the novelty of the song puts the mind in a more receptive state. This was concluded from the fact that the scores while listening to Indian music were the best and everyone claimed that they were hearing that song for the first time.

Since we asked the participants to carry out the same multiplication procedures three times in a row, they might have gotten used to the numbers. The use of tasks with comparable difficulty or the use of counterbalanced design was preferable^5)^. As a result, we could not accurately judge whether the music affected the time the participants took to complete the calculations or the practice did. To solve this problem, we could have asked them to do one set of multiplication problems each day or prepared three different kinds of calculation problems. In the memorization section, the participants admitted that some capital cities they wrote were ones they already knew, not ones they had memorized during the two minutes. The limitation also includes the number of participants. Because the number of participants in each task type was small, low reliability of the tests could have affected the instability of the results. The fact that the participants were nonequivalent groups also should be added as one of the limitations.

## Funding

No funding was received.

## Author contributions

RO, MS, SM, and RY planned the work and wrote the manuscript. RF supervised the work and revised the manuscript. All authors read and approved the final manuscript.

## Conflicts of interest statement

The authors have declared that no conflicts of interest exist.
